# Directed Communication between Nucleus Accumbens and Neocortex in Humans Is Differentially Supported by Synchronization in the Theta and Alpha Band

**DOI:** 10.1371/journal.pone.0138685

**Published:** 2015-09-22

**Authors:** Jörn M. Horschig, Ruud Smolders, Mathilde Bonnefond, Jan-Mathijs Schoffelen, Pepijn van den Munckhof, P. Richard Schuurman, Roshan Cools, Damiaan Denys, Ole Jensen

**Affiliations:** 1 Donders Institute for Brain, Cognition and Behaviour, Radboud University, Nijmegen, The Netherlands; 2 Department of Psychiatry, Academic Medical Center, Amsterdam, The Netherlands; 3 Max-Planck Institute for Psycholinguistics, Nijmegen, The Netherlands; 4 Department of Neurosurgery, Academic Medical Center, Amsterdam, The Netherlands; 5 Department of Psychiatry, Radboud University Medical Center, Nijmegen, The Netherlands; 6 The Netherlands Institute for Neuroscience, Royal Netherlands Academy of Arts and Science, Amsterdam, The Netherlands; Universiteit Gent, BELGIUM

## Abstract

Here, we report evidence for oscillatory bi-directional interactions between the nucleus accumbens and the neocortex in humans. Six patients performed a demanding covert visual attention task while we simultaneously recorded brain activity from deep-brain electrodes implanted in the nucleus accumbens and the surface electroencephalogram (EEG). Both theta and alpha oscillations were strongly coherent with the frontal and parietal EEG during the task. Theta-band coherence increased during processing of the visual stimuli. Granger causality analysis revealed that the nucleus accumbens was communicating with the neocortex primarily in the theta-band, while the cortex was communicating the nucleus accumbens in the alpha-band. These data are consistent with a model, in which theta- and alpha-band oscillations serve dissociable roles: Prior to stimulus processing, the cortex might suppress ongoing processing in the nucleus accumbens by modulating alpha-band activity. Subsequently, upon stimulus presentation, theta oscillations might facilitate the active exchange of stimulus information from the nucleus accumbens to the cortex.

## Highlights

- Both theta (4–7 Hz) and alpha (9–14 Hz) facilitate corticostriatal interactions.
- The nucleus accumbens communicates with cortical oscillations mainly in the theta-band.
- Alpha oscillations represent control from the neocortex over nucleus accumbens.
- Upon stimulus processing, the theta-band connectivity increases.

## Introduction

There is growing evidence for a key contribution of communication between striatum and neocortex in human cognition, attention and behavior. However, little is known about the neuronal dynamics supporting such cortico-striato-cortical communication. Recordings from deep brain electrodes implanted in humans for the treatment of psychiatric disease provide a rare opportunity to investigate such subcortico-cortical dynamics. Deep-brain stimulation of the nucleus accumbens, a part of the ventral striatum, has been successful in treatment of refractory-resistant obsessive compulsion disorder [1–3], major depressive disorder [4–9] and also drug addiction [10,11]. Earlier studies reported on theta (4–7 Hz) and alpha (9–14 Hz) band oscillations in the human nucleus accumbens [12–15], as well as top-down directed synchrony between nucleus accumbens and frontal electrodes in the low frequencies between 1 and 10 Hz [16]. However, none of these studies explored the differential roles of alpha and theta oscillations in communication between neocortex and the nucleus accumbens.

Anatomically, the nucleus accumbens receives input from different neocortical areas, mainly from temporal and prefrontal areas. Indirectly the nucleus accumbens projects back to the prefrontal cortex and parietal cortex via the globus pallidus, subthalamic nucleus and the thalamus [17–19]. Computational models by Frank & O’Reilly [20–22] propose that the striatum acts as a gatekeeper by deciding whether stimulus information should or should not be passed on to the prefrontal cortex. Moreover, the nucleus accumbens has recently been suggested to be involved in detection of visual information, and actively modulates the degree of neocortical frontoparietal connectivity [23]. In this study, we investigated whether the nucleus accumbens, as part of the ventral striatum, and the neocortex are employing theta and alpha band oscillations in anticipation and during processing of visual stimulation, and whether the nucleus accumbens contributes to frontoparietal connectivity in these two frequency bands.

## Materials and Methods

### Participants

Seven right-handed patients (one male diagnosed with chronic major depressive disorder, one male with cocaine and opiate addiction, and five female and one male patient with obsessive-compulsive disorder; 22–55 years of age).participated in the experiment. The experiment was approved by the local Medical Ethical Committee of the Academic Medical Center, University of Amsterdam. All patients provided written informed consent according to the Declaration of Helsinki and the local Medical Ethical Committee of the Academic Medical Center, University of Amsterdam prior to the experiment. All patients underwent surgery for implantation of deep brain electrodes in bilateral nucleus accumbens (NAc) between 2010 and 2012 (see [1,3] for more information about the exact procedure). The most ventral contact point was located in the core of the NAc, and the three other contact points were extending into the ventral part of the anterior limb of the internal capsule. Data from the male OCD patient was unsuited due to excessive movement during the experiment, and low signal-to-noise ratio particularly in intracranial electrodes.

### Stimulus presentation and experimental paradigm

#### Equipment

Stimulus presentation was performed using Presentation (Version 14.5; Neurobehavioural Systems, Inc.) and a laptop (HP 6730b) computer screen on a 15.4 inch display at a resolution of 1024 by 768 pixels (refresh rate of 60 Hz). The distance from the screen to the participants was kept around 60 cm.

#### Paradigm

**Fig 1:**
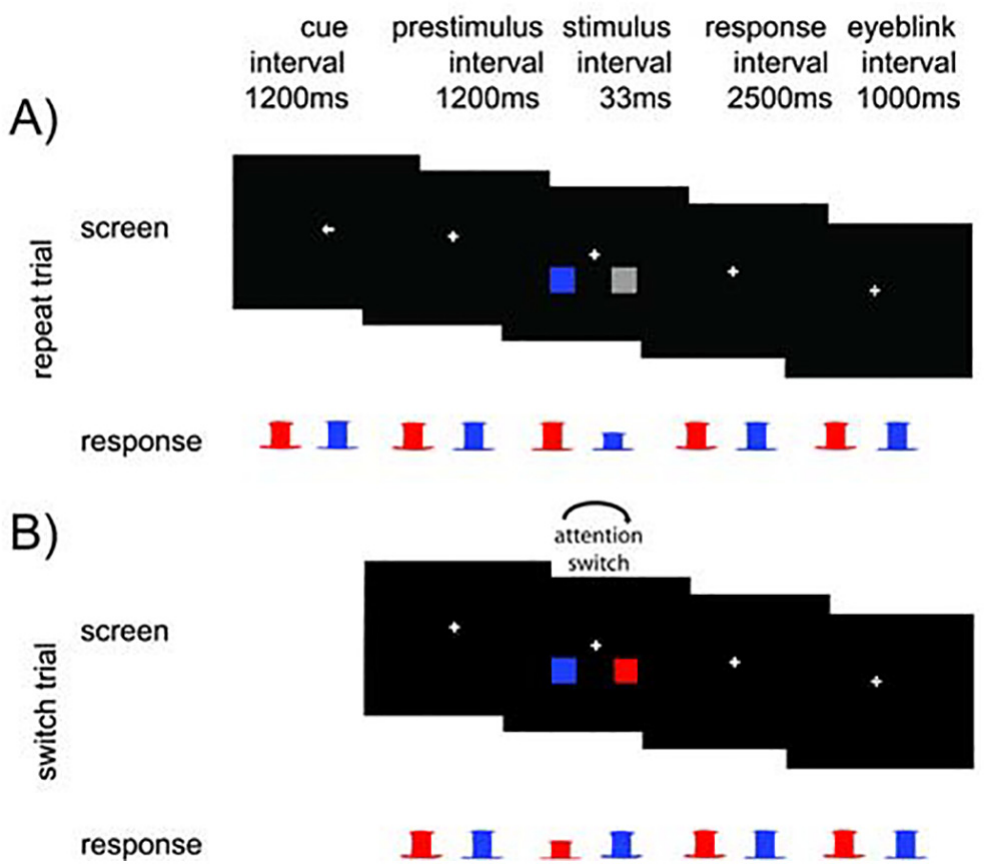
The paradigm. The attended side was initially indicated by a cue. Subjects had to fixate at the central cross and by button press indicate the color of the attended squares (left button for red and right button for blue). The 1200 ms prestimulus period was followed by the colored stimuli flashed for 33 ms. Subjects had to respond within 2500 ms. If there was a color change in the square of the unattended hemifield, attention had to switch to that direction (‘switch-trial). After the response there was a 1000 ms window for eye blinking. A Example of an explicit cue followed by a repeat trial. The subject had to covertly attend to the left and subsequently report the color of the stimuli by pressing the corresponding button (here: blue, right button). B Example of a switch trial. In the previous repeat trials, the subject had to attend to the left, because of the initially shown spatial cue. Upon stimulus presentation, the subject correctly switched attention and indicated so by reporting the color of the stimulus at the formerly unattended side (here: right, red color). If the subject responded according to the formerly attended side (here: left, blue), the switch trial would repeat up to four times. Repetitions of switch trials were removed from the analysis. If the subject did not switch after the fourth consecutive switch trial, another explicit spatial cue pointing to the formerly unattended side was presented (here: a rightward pointing arrow).At the beginning of each block, subjects were explicitly cued to which side to attend. From then on, the attended side was determined by stimuli properties alone. A central fixation point was presented during the entire experiment. Colored squares were flashed 1200ms after the beginning of each trial for about 33 ms (two frames = 2/60Hz). These stimuli were presented with six degrees eccentricity and two degrees lower than the fixation cross (measured from the fixation cross to the center of the stimuli). The squares were two degrees wide.

We adapted a covert attention switching paradigm also described in [24], see Fig 1. Squares were flashed on each side and subjects had to report the color of the attended square by a button press. When subjects detected a color change at the unattended side (signaling a *switch trial*), they had to report the color of the unattended square (but not the currently attended square) and switch attention to the unattended side in future trials.

Subjects had to report the color of the square on the attended side by pressing a button with their left (for red) or right hand (for blue). On the unattended side, the square was either grey (*repeat trials*) or colored in blue or red (*switch trials*). Subjects had to respond within 2500 ms. After responding, the fixation cross turned grey, indicating that the subject could blink or move the eyes in a 1000 ms period. Then the fixation cross turned white again indicating the start of the next trial. Subjects had to keep attention to one hemifield (*repeat trial*) and report the color of the square on that side until they detected a colored stimulus in the unattended hemifield (switch stimulus). The switch stimulus was detected if the color of the unattended target was correctly reported (*detected switch trial*). Subjects then had to keep attending the formerly unattended hemifield until a next switch trial was detected. If a subject failed to detect the switch stimulus (*undetected switch trial*), it was repeated with a random color (blue or red) up to four times. The probability of a switch trial was increasing with the number of trials since the last switch trial, but is not of further importance for this study (see [24] for more information).

#### Stimulus intensity

In order to make the task sufficiently difficult, the intensity of the stimuli was varied across subjects and early trials in an adaptive staircase-like procedure on a 20-step scale (1:darkest; 20:brightest), starting at 10 for both the neutral and the colored stimuli. This was done in the first block (i.e. until the 15th *detected* switch trial). Trials from this block were not included in the later analyses. The intensities of the neutral and colored stimuli were modulated according to different criteria. Repeat trials should be sufficiently demanding while keeping response errors as low as possible. Therefore we reduced the brightness of the colored stimuli to a level in which the subject could perform the discrimination with no errors. The color intensity was reduced by one step after each *detected switch trial* and error-free performance. After a response error to repeat trials, the intensity was increased by one step again. The intensity of the neutral stimulus was adapted to manipulate the difficulty of switch trials. A large intensity difference between colored and neutral stimuli makes detection of switch trials easier (pop-out effect), whereas a similar intensity results in a harder task and less detected switch trials. We aimed at a correct response rate to switch trials between 25% and 75%. After a detected switch trial, the neutral stimulus was increased in intensity by one when less than 25% of all switch trials were detected. The intensity was decreased by one step when more than 75% of all switch trials were detected. The twenty levels of stimulus luminance were visually and mathematically matched according to the CIELAB specifications [25,26]. Note that the adaptation procedure was only applied in the first block until the 15^th^ detected switch trial.

#### Instructions

Prior to the experiment, participants received written and verbal task instructions. Subjects were instructed to prioritize accuracy rather than speed, but were informed that they should respond within 2500 ms. Subjects were instructed to detect the color at the cued side, but switch attention to the uncued side if the color at that side turned from grey to either blue or red. Subjects were informed that they would receive no response feedback. The instructions did not inform about other task properties. After the instructions, subjects had to complete a short tutorial on the computer, which explained the paradigm and introduced the stimuli. Afterwards the experimental task started. In our analysis, as already reported above, we discarded all trials up to the first experimental break.

### Data acquisition

The ongoing brain activity was recorded using a 64-EEG-electrode recording system (Advanced Neuro Technology B.V.) at a sampling rate of 512Hz following the international 10–10 system. Eight channels were used to record the deep brain electrodes near the nucleus accumbens (four electrodes per lead), four to derive horizontal and vertical eye-movement, leaving 52 Ag/AgCl electrode distributed evenly on the scalp while leaving out areas covered by post-surgery bandages. Data was online common average referenced and offline re-referenced to the common electrodes across patients. For the final analysis, only electrodes that were common for all patients were included. These were the following 42 electrodes: AF7, AF8, C1, C2, C3, C4, C5, C6, CP1, CP2, CP3, CP4, CP5, CP6, CPz, Cz, F1, F7, F8, FC5, FC6, FCz, FT7, FT8, Fp1, Fp2, Fpz, Fz, O1, O2, Oz, P1, P2, P3, P4, P5, P6, PO3, PO4, PO7, POz and Pz. Purely for visualization purposes, missing electrodes were interpolated using a spherical spline interpolation [27].

### Data preprocessing and analysis

#### Preprocessing

The EEG data were analyzed using the Matlab-based FieldTrip toolbox, developed at the Donders Institute for Brain, Cognition and Behaviour [28]. We first visually inspected activity in all electrodes and rejected electrodes with a low signal-to-noise ratio. Then, we re-referenced all electrodes to a common average reference. Muscle and ocular artifacts were detected in a semi-automatic fashion, which included visual inspection and trial rejection based on variance and other measures as implemented in FieldTrip. We rejected all trials with an artifact in the interval -1000 ms to 500 ms relative to stimulus onset.

To estimate activity of the left and right nucleus accumbens, we computed the bipolar derivative of the most ventral and the second most ventral deep brain electrode (which was located in the core of the NAc) for the left and right lead, respectively. We will refer to these as left and right nucleus accumbens. For the main manuscript, only data from the left nucleus accumbens were used. Thus the term “nucleus accumbens” refers to the bipolar derivative of the electrodes in the core of the left nucleus accumbens and the most ventral electrode in the ventral part of the anterior limb of the internal capsule. Data from the right contact points showed generally more noise in most patients, and in one patient no data for the right nucleus accumbens was recorded. We therefore decided to conduct no further analysis on the data from the right nucleus accumbens.

#### Spectral analysis

We computed Fourier coefficients from 0 Hz to 256 Hz for three time windows: the whole trial window from -1000 ms to 500 ms relative to stimulus onset, the prestimulus window from -500 ms to 0 ms, and the poststimulus window from 0 ms to 500ms. All data windows were zero-padded by 4500 ms to increase the spectral resolution and thereby smooth the Fourier estimates to avoid sudden transitions, especially around the 50 Hz noise-band. This resulted in a frequency resolution of 0.16 Hz for the whole trial window and 0.2 Hz for the pre- and poststimulus windows. For visualization, we show only the content from 2 to 18 Hz.

We computed the time-frequency representations (TFRs) of power from 0 to 256 Hz (0.5 Hz increments) for each trial from a -1000 ms to 500 ms interval around the stimulus onset. Spectral content was estimated using a 500 ms time window, which was multiplied with a Hanning window prior to applying a fast Fourier transform. For visualization, we only show the low frequencies up to 30Hz.

#### Coherence analysis

We compute the squared coherence for each combination of the nucleus accumbens and extracranial EEG electrodes. Coherence *C*_*xy*_ *(f)* between two signals *x* and *y* is computed using the power spectra of both electrodes *S*_*xx*_ *(f)* and *S*_*yy*_ *(f)* and their cross-spectrum *S*_*xy*_ *(f)* by the following equation [29]:

$$C_{xy}\left(f\right)=\frac{{\left|S_{xy}(f)\right|}^{2}}{\left(S_{xx}\left(f\right)S_{yy}\left(f\right)\right)}$$ (1)

Coherence is the frequency domain version of the linear correlation coefficient, and quantifies the consistency of the phase difference and amplitude correlations across observations. A coherence *C*_*xy*_ *(f)* of *0* indicates that the signals *x* and *y* have no consistent linear phase relationship, whereas a value of *1* indicate that the two signals are fully phase-coherent.

#### Non-parametric Granger causality

Granger causality in the time-domain is well defined and has been well-established throughout the last century [30,31]. From the time-domain formulation of Granger causality, one can directly infer a formulation for non-parametric Granger causality in the frequency domain [32–35]. We summarize the most important aspects of this derivation here and outline its relation to coherence as explained above.

A signal *x* is said to “Granger cause” signal *y* if the future of signal *y* can be better explained by incorporating knowledge about the past of signal *x*, above and beyond the prediction of the future of signal *y* based on its own past alone.

Total interdependence *f*_*x*,*y*_ *(f)* between of two signals *x* and *y* is computed by their power spectra *S*_*xx*_ *(f)* and *S*_*yy*_ *(f)* and their cross spectra *S*_*xy*_ *(f)* as

$$f_{x,y}\left(f\right)=\text{ln}\;\left(\frac{S_{xx}(f)S_{yy}(f)}{\left|S(f)\right|}\right)$$ (2)

where *ln* denotes the natural logarithm, and *S*(*f*) = *S*_*xx*_(*f*)*S*_*yy*_(*f*) − *S*_*xy*_)(*f*)*S*_*yx*_(*f*). From Eqs (1) and (2), one can infer that

$$f_{x,y}\left(f\right)=-\text{ln}(1-C_{xy}(f))$$ (3)

Furthermore, total interdependence can be decomposed into three different causality terms:

$$f_{x,y}\left(f\right)={\text{f}}_{\text{x}\to \text{y}}+{\text{f}}_{\text{y}\to \text{x}}+{\text{f}}_{\text{x}\cdot \text{y}}$$ (4)

Where *f*_*x→y*_ denotes the causal influence from signal *x* to signal *y*, *f*_*y→x*_ denotes the causal influence from signal *y* to causal *x* and *f*_*x·y*_ denotes the *instantaneous causality*, i.e. contributions by non-linear interactions between x and any contributions exogenous to signal *x* and *y* or by common input from a third signal.

From the derivations of [32–33] it follows that

$$f_{x\to y}(f)=-\text{ln}\;\left(\frac{S_{xx}(f)}{{\tilde{H}}_{xx}\Sigma_{2}{{\tilde{H}}_{xx}}^{*}}\right)$$ (5)

$$f_{y\to x}(f)=-\text{ln}\;\left(\frac{S_{yy}(f)}{{\tilde{H}}_{yy}\Gamma_{2}{{\tilde{H}}_{yy}}^{*}}\right)$$ (6)

where ***Σ***_*2*_ and ***Γ***_*2*_ denote the variance of the noise-term in the bivariate model of signal *x* and *y*, respectively (i.e. can be obtained from their noise covariance matrix ***Σ***), ${\tilde{H}}_{xx}$ denotes the spectral transfer function of signal *x* after normalization, and * the complex conjugate. The instantaneous causality is thus the difference between the total interdependence of x and y and the sum of the two causality terms *f*_*x→y*_ and *f*_*y→x*_. Note that the spectral representations *S*_*xx*_ and *S*_*yy*_ can also be fully estimated by the spectral transfer function *H*_*xx*_ and *H*_*yy*_ and the noise-covariance matrix ***Σ***. For a more thorough derivation see [32–33].

The normalized spectral transfer function ${\tilde{H}}_{xx}$ of signal *x* can be derived using a matrix factorization method on the spectral representation of signal *x* [36]. Note that the spectral representations *S*_*xx*_ and *S*_*yy*_ can also be fully estimated by the normalized spectral transfer function ${\tilde{H}}_{xx}$_*xx*_ and ${\tilde{H}}_{yy}$_*yy*_ and the noise-covariance matrix ***Σ***.

#### Statistical analysis

Statistical significance of the coherence of the neural data was assessed using a non-parametric cluster-based permutation test [37,38]. In the cluster-based permutation test, notational significant clusters in space are detected using a parametric test-statistic. We z-transformed the coherence values to correct for the variable number of degrees of freedom (trials numbers) across patients. In accordance to [38], if there are more than 20 degrees of freedom and if the squared population coherence is between 0.4 and 0.95, then tanh^−1^ (*C*_*xy*_(*f*)) is approximately normal distributed with mean tanh^−1^ (*C*_*xy*_(*f*)) − 1/(*df* − 2) and variance 1/(*df* − 2). Thus, a standard z-transformation can therefore be performed to obtain Z_xy_ as follows:

$$Z_{xy}=\frac{{\text{tanh}}^{-1}\left(\left|C_{xy}(f)\right|\right)-1/\left(df-2\right)}{\sqrt{1/\left(df-2\right)}}$$ (7)

Note that although above mentioned requirements are not met in our data, this is no reason for concern as we applied the here described nonparametric test. This transformation was only done to account for the different amount of degrees of freedom across patients.

We compared the observed z-transformed coherence Z_xy_ with a surrogate distribution of maximal z-transformed coherence cluster sizes. One instance of the surrogate distribution was computed as follows: For each patient, we randomly shuffled trials in the intracranial data, while remaining the order of trials of the EEG data. Then, we averaged over time and frequency of interest (here -1s to 0.5s, and 4 Hz to 14 Hz). After we computed the z-transformed coherence Z_xy_ for each patient of the shuffled data, we computed grand-average z-transformed coherence across patients. If the summed cluster size exceeded 3.92, which is twice the threshold at alpha = 2.5%, we defined the cluster as a notational cluster. This was conducted 500 times to obtain a surrogate distribution. We then compared the size of notational clusters in the original observation with the size of notational clusters found in the surrogate distribution. We assessed significance in the original observation by a 5%-alpha level, i.e. 95% of the surrogate data had to contain smaller cluster sizes than those in the original observation for the test to be statistically significant. A cluster-based permutation test controls for multiple comparisons as only one comparison is made (the cluster size of the actual observation versus the surrogate distribution of cluster sizes).

Further statistical tests were performed by analysis of variance (ANOVA) and post-hoc paired t-tests. As parametric tests require normally distributed data, we log-transformed data prior to performing ANOVA or paired t-tests if data were not normally distributed as assessed by a Kolgomorov-Smirnov test. Effect sizes were computed by assessing η^2^ = SS_effect_/SS_total_ and subsequently transformed to Cohen’s f (see e.g. [39]) as

$$f=\;\sqrt{\frac{\eta^{2}}{1-\eta^{2}}}$$ (8)

While η^2^ give an estimate of the explained variance, Cohen’s f is a standardized measure of effect sizes and widely used throughout different fields (psychometric testing, psychological tests, neuroscientific investigations, etc.)

In addition, we performed a permutation test on Granger directionality estimates. For each of the six subjects, we obtain one Granger estimate for the cortex-to-nucleus accumbens direction and one Granger estimate for the nucleus accumbens-to-cortex direction. For each subject, we subtracted these two directionality estimates to obtain the difference. In line with permutation testing, we permuted the directionality estimates for each subject, which effectively was achieved by swapping the sign of the difference in directionality. With two possible directions in six subjects, there are in total 2^6^ = 64 permutations possible, of which one is the original observation. We computed the grandaverage difference in directionality for the original observation and for all other 63 permutations. Then, we assessed how many permutations resulted in a more extreme grandaverage difference in directionality with a binomial test. This number divided by the total number of permutations results in a p-value. We assessed statistical significance at an alpha level of 0.05.

## Results

We investigated data from six patients (four with OCD, one with major depression disorder and one with drug addiction) with four-contact deep brain stimulation electrodes implanted bilaterally in the ventral anterior internal capsule, with the deepest of the four electrode contact points situated in the nucleus accumbens. Prior to deep brain stimulation, the deep brain electrodes were externalized, and we simultaneously recorded data from the intracranial electrodes and the scalp EEG. All the reported results are from the left nucleus accumbens. Data from the right nucleus accumbens were more noisy and incomplete in some patients. Patients performed a demanding attention task without any response feedback or reward, which did not include symptomatic related stimuli. Patients had to continuously attend one hemifield and report the color of shortly presented boxes, until a stimulus change at the unattended side occurred. The onset of the stimuli was predictable: after a 1200 ms prestimulus period, two boxes were shortly presented for 33 ms. Patients had to discriminate the color of the box on the attended side, while the unattended box was grey. In some trials, the box at the unattended side was colored as well, and the subjects had to switch attended side and report the color of the box presented on that side. From then on the patients had to attend that side. In this study we specifically investigated how theta-band and alpha-band oscillations supported the functional connectivity between the nucleus accumbens and the cortex, both in anticipation of and in response to the stimulus.

### Behavioral results

The total duration of the experiment was 30 minutes. On average, the participants completed 446±(SD) 59 trials, of which 224±34 trials were on attention to the left and 223±26 on attention to the right. 120±29 trials were *switch trials*, and 326±39 trials were *repeat trials*. Participants responded correctly in 96±2% of all repeat trials, with no significant difference between attention left and attention right repeat trials (t(5) = -1.7, p = 0.16). Participants detected switch trials correctly in 74%±18% (*switch rate*) of all *switch trials*. Notably, participant 4 (the depressed patient) had a switch rate of only 48%, while all other participants had a switch rate between 76% and 94%. There was no significant difference between switch trials to the left or the right hemifield within participants (t(5) = 0.66, p = 0.54). These rates are comparable with the one we reported in [24], where healthy participants performed the same task in a more demanding version (further eccentricity of the stimuli) and correctly responded to 91±4.6% of all repeat trials and 64.2±19% of all switch trials.

Participants responded to repeat trials in 758±185 ms, with no significant difference between attention left and right hemisphere (t(5) = 0.54, p = 0.6). In switch trials, participants responded in 1119±278 ms with no significant difference between switch trials to the left and right hemifield (t(5) = 0.19, p = 0.86) and no significant difference between correct and incorrect responses to switch trials (t(5) = 0.09, p = 0.93).

### Spatial topography of coherence

**Fig 2:**
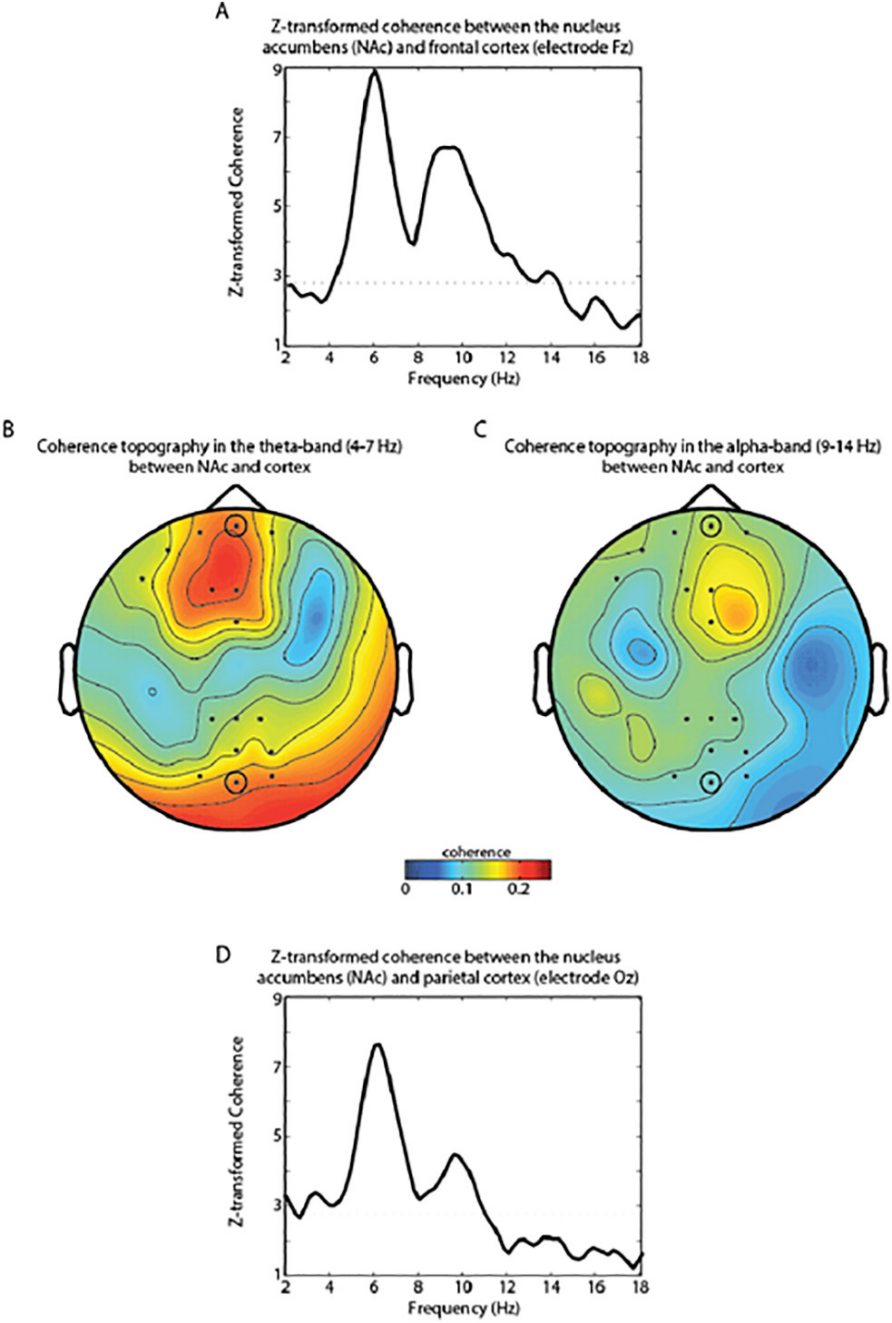
Coherence between the nucleus accumbens (NAc) electrodes and the scalp EEG. **A** Z-transformed coherence spectrum between nucleus accumbens and frontal scalp electrode Fz. The horizontal dotted line (Z = 2.8) indicates the threshold of the coherence calculated from data shuffled over trials (see Methods). Coherence values between 4 Hz and 14 Hz are above this threshold. Electrodes Fz and Oz are circled. **B** Coherence in the theta-band showed a strong frontal topography and a weaker parieto-occipital topography. **C** Coherence in the alpha-band was strongest around frontal electrodes. The *’s indicate scalp electrodes that were significantly coherent between 4–14 Hz with nucleus accumbens when controlling for multiple comparisons over electrodes and frequency bins using a cluster randomization approach. A strong frontal and a weaker parietal cluster emerged. **D** Example of coherence in the parietal cluster. The Z-transformed coherence spectrum between nucleus accumbens and occipitoparietal scalp electrode Oz is depicted.

First, we investigated frontostriatal connectivity by computing coherence between the nucleus accumbens and the scalp EEG electrodes for the interval from -1000 ms to 500 ms relative to stimulus onset [29]. For all connectivity analyses, we pooled data of all trial types. Fig 2A shows two clearly distinct peaks, one in the theta-frequency range from 4 to 7 Hz and the other one in the alpha-frequency range from 9 to 14 Hz. The data were z-transformed to normalize over subjects prior to averaging. To assess whether the observed coherence was statistically significant, we shuffled the data over trials and recalculated the coherence and used the maximum coherence value in this surrogate distribution as a threshold. We identified coherence values between 4 Hz and 14 Hz to be above this threshold calculated from the shuffled data. To statistically assess spatial specificity of this frequency-band, we performed a cluster-based permutation with surrogate data to identify in what scalp EEG electrodes the coherence with nucleus accumbens in the frequency range from 4 to 14 Hz was strongest [38]. The permutation test controls for multiple comparisons over electrodes and frequency bins. There was a highly significant difference between the observed and the surrogate data (p<0.01), with one cluster over the frontal cortex and one cluster over parietal cortex, which we subsequently defined as regions of interest (ROIs). Figs 2B and 1C show topographic representations of the coherence in the theta- and the alpha-band. Coherence in the theta-band was in general higher than in the alpha-band, and coherence in the frontal ROI was generally stronger than the parietal ROI (see also Fig 2D).

### Spectrotemporal dynamics of the oscillatory coupling

**Fig 3:**
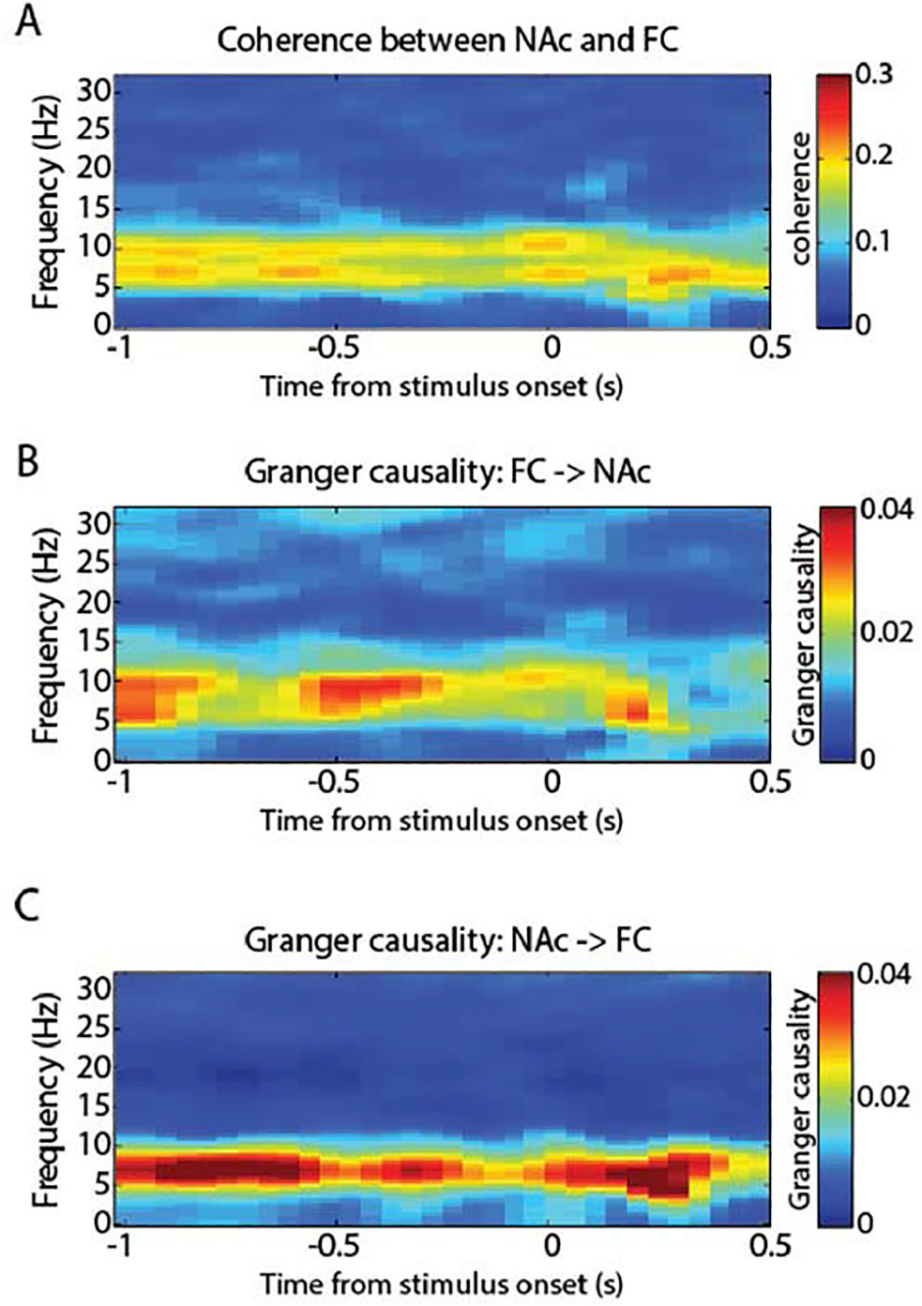
Time-frequency dynamics of cortico-striatal interactions between nucleus accumbens (NAc) and frontal cortex (FC). **A** Time-frequency coherence spectrum. In the prestimulus period (-1000 ms to 0 ms), both alpha and theta band coherence were strong. After stimulus onset (0 ms), alpha band coherence decreased while theta band coherence increased. **B** Granger causality from frontal electrodes to nucleus accumbens was highest in the alpha range (9–14 Hz) and diminished upon stimulus processing (after 0 ms). **C** Granger causality from nucleus accumbens to frontal electrodes was strongest in the theta range and increased upon stimulus processing (after 0 ms).

Subsequently, we explored the spectrotemporal dynamics of the coherence in response to the stimulus. To this end we conducted a time-resolved coherence analysis from -1000 ms to 500 ms relative to stimulus onset using a sliding window technique with Hanning-tapered windows of 500 ms length). In Fig 3A we show the coherence between electrodes in the frontal cluster and the nucleus accumbens electrodes (marked in Figs 2C and 1D). In the prestimulus interval, we observed high, sustained coherence in the theta- and alpha-bands. During stimulus processing, around 300 ms after stimulus onset, alpha-band coherence appeared weaker whereas coherence in the theta-band became stronger. This speaks to the functional relevance and different functional roles of theta- and alpha-band functional connectivity during stimulus processing.

In Fig 3A, alpha-band coherence appeared to become weaker whereas coherence in the theta-band became stronger around 300 ms after stimulus onset. When comparing the prestimulus interval (-500ms to 0ms) with the poststimulus interval (0ms to 500ms), these changes were, however, not significant, neither the theta-band increase (around Fz, t(5) = 1.31, p = 0.25), nor the alpha-band decrease (around Fz, t(5) = -1.22, p = 0.28). When posthoc pre-selecting the 300ms time point, however, we found that theta-band activity was significantly higher than during stimulus onset (comparing coherence from 4–7Hz at 0ms vs. 300ms; t(5) = 2.91, p = 0.03). No effect at this time point for the alpha-band (9–14Hz) was found (t(5) = -1.96, p = 0.11).

To further investigate the functional connectivity, we conducted a time-resolved Granger causality analysis with the same parameters that we used to compute the temporal coherence spectrum [33–35]. Fig 3B shows the spectrotemporal dynamics of the Granger causality measure applied to frontal EEG and intracranial nucleus accumbens electrodes. In order to assess whether the resulting frequency-bands of the Granger causality analysis in the two directions were different from each other, we assessed the peak frequency of the Granger values in each subject in the range between 4 Hz and 14 Hz. We compared peak frequency from NAc to cortex (6.16 Hz +/- 0.6 Hz) to the peak frequency from cortex to NAc (8.9Hz +/- 1.7Hz) and found them to be significantly different from each other (t(5) = 3.42, p = 0.02). Consistent with contemporary literature and the coherence results, the lower of these two frequency peaks is in the theta-band between 4 Hz and 7 Hz, and the higher of the two peak frequencies is around the alpha-band, defined between 9 Hz and 14 Hz.

In Fig 3B, we further observe that in anticipation of the stimulus, sustained activity in the NAc predicted alpha band-activity of the cortex (from here on referred to as ‘granger-causal influence’ or simply ‘connectivity’). Upon stimulus processing, the alpha-band connectivity became weaker. In the reverse direction (Fig 2C), we found that theta-band activity from the NAc predicted activity around the frontal cortex in anticipation of the stimulus, which further increased during processing of the stimulus. Results for the parietal ROI are comparable, therefore not shown.

One might argue that modulation of coherence and Granger causality measures can be explained by concurrent changes in power. Therefore, we compared power in the theta and alpha bands between the prestimulus (-500 ms to 0 ms relative to stimulus onset) and poststimulus intervals (0 ms to 500 ms relative to stimulus onset). There was no statistically significant changes in power between these intervals in the theta-band, neither in the nucleus accumbens (t(5) = -1.12, p = 0.31), nor the frontal cluster (t(5) = 1.21, p = 0.28) and a near significant theta power decrease in the parietal cluster (t(5) = 2.27, p = 0.07). We conclude that the increase in coherence at the theta-band in response to the stimulus cannot be explained by a concurrent theta power increase in the cortex nor the nucleus accumbens. In the nucleus accumbens, there was no statistically significant change in alpha-power in response to the stimulus (t(5) = 1.00, p = 0.36), but there was a significant alpha-power decrease in the frontal ROI (t(5) = 3.92, p = 0.01) and in the parietal ROI t(5) = 3.1, p = 0.03). Thus, we cannot exclude that the decrease in alpha coherence in response to the stimulus is affected by the strong decrease in cortical alpha power.

To access the robustness of the modulations in Granger causality we conducted a repeated-measures ANOVA with four factors: *region of interest* (frontal and parietal EEG electrodes), *time* (the -500–0 ms prestimulus window and the 0–500 ms poststimulus window), *frequency* (the 4–7 Hz theta band and the 9–14 Hz alpha band) and *directionality* (nucleus accumbens to neocortex and neocortex to nucleus accumbens). Granger estimates were log-transformed to approach a normal distribution (Kolgomorov-Smirnov test; non-log transformed data: p<0.001; log-transformed data: p>0.5). In the following we report only significant, or near-significant results from the ANOVA (p<0.1); for all results see Table 1. The ANOVA showed a main effect of frequency (F(1,5) = 7.68, p<0.00393, Cohen’s f = 0.39), caused by Granger causality in the theta-band generally being stronger than in the alpha-band. There was a trend towards a main effect of time (F(1, 5) = 4.78, p = 0.08, Cohen’s f = 0.17).

**Table 1:** Granger causality ANOVA results.

Factor	F-value	p-value
Time	4.78	0.0805
Channel	0.26	0.6287
Frequency	7.68	0.0393
Directionality	0.09	0.781
Time * Channel	1.83	0.2339
Time * Frequency	20.92	0.006
Time * Directionality	0.01	0.9453
Channel * Frequency	3.89	0.1055
Channel * Directionality	0.12	0.7385
Frequency * Directionality	9.68	0.0265
Time * Channel * Frequency	1.24	0.3155
Time * Channel * Directionality	2.6	0.1676
Time * Frequency * Directionality	1.85	0.2316
Channel * Frequency * Directionality	0.35	0.5781
Time * Channel * Frequency * Directionality	0.91	0.3851

**Fig 4:**
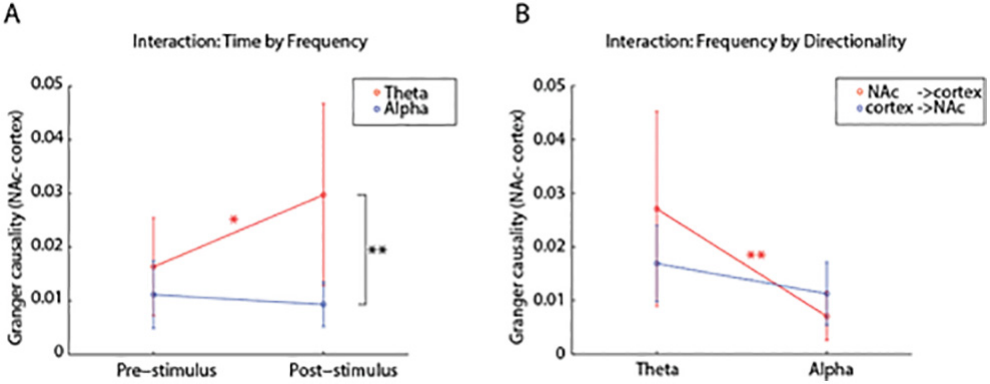
Significant interaction effects of the factorial ANOVA on the Granger estimates. **A** The *time* by *frequency* interaction (F(1, 5) = 20.92, p<0.01, Cohen’sf = 0.17) indicates that there was a significant theta band increase poststimulus compared to prestimulus Granger causality (t(5) = 3.52, p<0.05). No such change was present in the alpha-band. In the poststimulus window, Granger causality in the theta band was significantly stronger than in the alpha band (t(5) = 4.27, p<0.01). **B** The ANOVA showed a significant frequency by directionality interaction (F(1, 5) = 9.68, p<0.05, Cohen’s f = 0.17). In post-hoc tests, we found that the granger-causal influence from nucleus accumbens on cortex was stronger in the theta-band than in the alpha-band (t(5) = 4.84, p<0.01). In contrast, there was no significant difference in Granger causality in the alpha-band between cortex-to-nucleus accumbens and nucleus accumbens-to-cortex direction (t(5) = 1.75, p = 0.14). However, for five out of six subjects Granger estimates in the alpha-band were stronger for the cortex-to-nucleus accumbens direction than the other way around (see Fig 5). Data are represented as mean ± SEM. * = p<0.05, ** = p<0.01. = p<0.01.

In addition, we found a significant *time* by *frequency* interaction (F(1, 5) = 20.92, p = 0.006, Cohen’s f = 0.17, see Fig 4A). A posthoc analysis revealed that there was a significant theta-band Granger causality increase in the poststimulus interval compared to the prestimulus interval (t(5) = 3.52, p = 0.017). In contrast, Granger causality in the alpha band showed no significant change in time (t(5) = -0.08, p = 0.94). Furthermore, theta-causality in the post-stimulus interval was stronger than alpha-causality (t(5) = 4.27, p = 0.008). This suggests that stimulus processing is associated with increased connectivity in the theta-band between nucleus accumbens and neocortex.

Crucially, we found a significant interaction between *frequency* and *directionality* (F(1, 5) = 9.68, p = 0.00265, Cohen’s f = 0.17; see Fig 4B). Posthoc t-tests revealed that the difference in theta-band Granger causality between nucleus accumbens to cortex and cortex to nucleus accumbens was larger than the same difference in the alpha-band. Granger causality from nucleus accumbens to cortex was significantly stronger in the theta-band than in the alpha-band (t(5) = 4.84, p = 0.005), indicating that theta-oscillations represent the primary granger-causal influence from nucleus accumbens to cortex.

**Fig 5:**
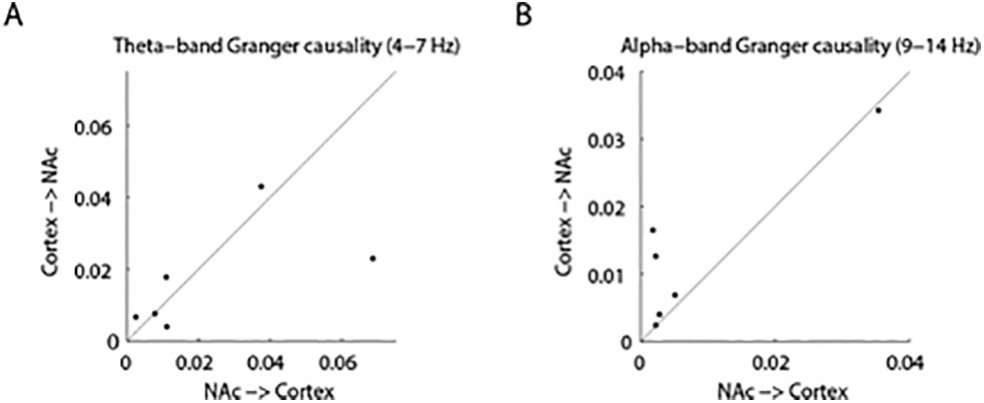
Granger causality values for the theta- and the alpha-band in the two directions calculated for the full time interval (-1 to 0.5 s). The solid line indicates the main diagonal to help illustrating in how many participants the Granger results were stronger in the one compared to the other direction A In half of the subjects, theta-band oscillations were stronger from cortex to nucleus accumbens (NAc) than the other way around. B Alpha-band oscillations were stronger from the cortex to the nucleus accumbens than from nucleus accumbens to the cortex for five out of six subjects (permutation test across subjects, p<0.05). This indicates that cortical alpha-band activity granger-cause activity in the nucleus accumbens.

In addition, we found that alpha-band activity from the cortex to NAc provided more predictive information than in the reverse direction from nucleus accumbens to the cortex in five out of six patients, which indicates that cortical alpha-band oscillations are granger-causing alpha-oscillations in the nucleus accumbens. Using conventional posthoc t-tests, this difference was, however, not significant (t(5) = 1.75, p = 0.14). The lack of statistical significance might be explained by the low number of subjects and by the low frequency resolution (2Hz) due to the 0.5s time windows, which results in spectral leakage between the theta- into the alpha-band. To further investigate the directional-specificity of the alpha-band, we analyzed the whole trial time-window from -1s to 0.5s using a permutation test. We performed a permutation test of Granger causality difference in the alpha-band across participants. For each participant, we computed the average Granger causality in the alpha-band in the whole time-window (-1 s to 0.5 s) between the nucleus accumbens and the cortex (i.e. the electrodes in two ROIs) and in the reverse direction (see Fig 5). Then, we subtracted the values for the two directions from one another. We created a permutation distribution by swapping the Granger estimates of the directionalities within subjects and performed the formerly mentioned procedure for all 64 (= 2^6^) possible permutation. We found that the average directionality difference was more extreme for only two out of the 64 permutations than in the actual observation (one-sided test; p<0.05). The same procedure for the theta band did not reveal any significant directionality preference (one-sided test; p>0.05). We therefore conclude that connectivity in the alpha-band reflects primarily communication from the cortex to the nucleus accumbens.

### The nucleus accumbens mediates long-range cortical communication

**Fig 6:**
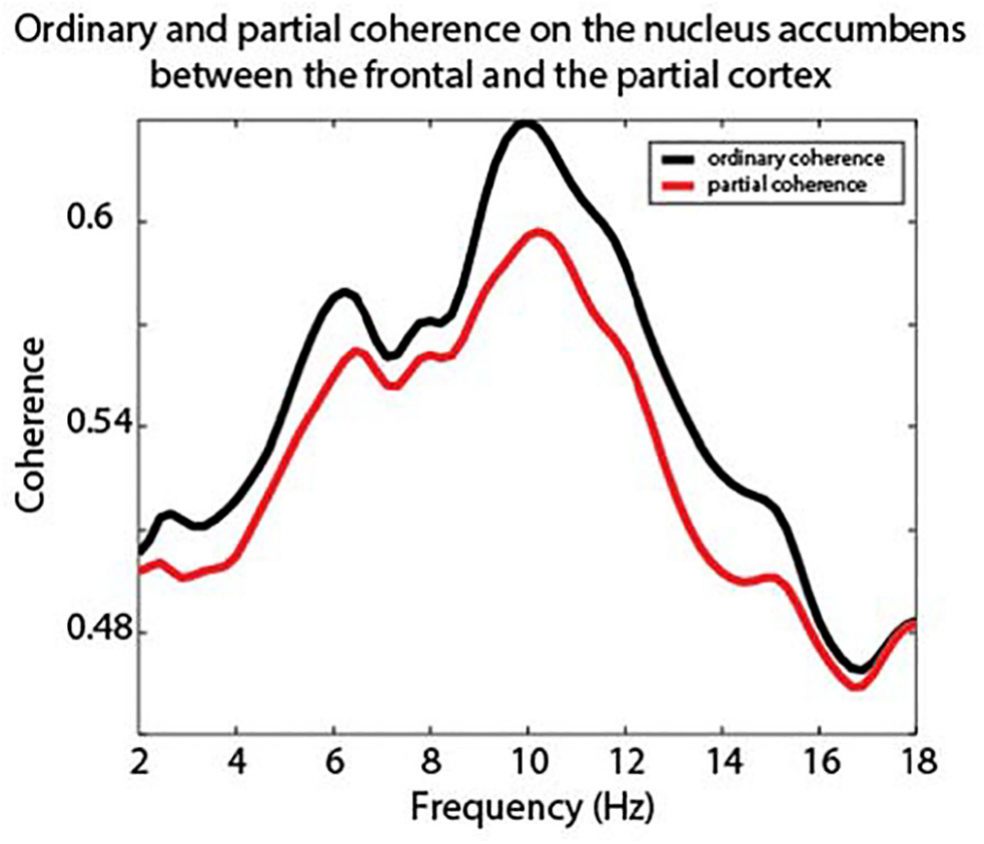
Partial coherence results. Ordinary coherence between the frontal and parietal cortex showed clear peaks in the theta and alpha frequency band. When partializing out the coherence from the nucleus accumbens, the frontoposterior coherence in the 9–14 Hz alpha-band (t(5) = 3.00, p = 0.03) decreased significantly.

Recently it has been argued that subcortical regions, e.g. the pulvinar, have an impact on large scale cortical communication [40,41]. Recent findings by van Schouwenburg, den Ouden & Cools [23,42] showed that the striatum has a modulatory influence on frontoposterior connectivity. Therefore, we investigated whether frontoposterior coherence in the theta and alpha-band is influenced by nucleus accumbens activity. We did this by considering the fronto-parietal coherence in relation to the contribution from the nucleus accumbens. Partial coherence helps explaining how the connectivity pattern between two sources is influenced by a third source [43,44]. In line with the findings by van Schouwenburg and colleagues, we hypothesized that if the nucleus accumbens is coordinating frontoposterior connectivity, then partializing out the contributions of the nucleus accumbens will reduce the fronto-parietal coherence. While the frontal and occipital cortex coherence was relatively strong in all frequency bands due to volume conduction, we still observed two peaks around the theta (4–7 Hz) and the alpha-band (9–14Hz; Fig 5, black line). When partializing out the contributions of the nucleus accumbens (Fig 6, red line), we found that coherence in the alpha-band decreased significantly (t(5) = 3.00, p = 0.03), whereas this was not the case for theta band coherence (t(5) = 1.84, p = 0.12). Consistently, five out of six subjects showed a decrease in alpha-band coherence, whereas four out of six showed a decrease in theta-band coherence. This finding corroborates the idea that the striatum facilitates long-range cortical communication between the frontal and posterior cortex.

## Discussion

We investigated how connectivity in corticostriatal networks is implemented by neuronal oscillations in the theta- (4–7 Hz) and alpha-band (9–14 Hz). For the first time, we showed bi-directional temporospectral dynamics between the nucleus accumbens and the neocortex in humans. First, we showed that the nucleus accumbens controlled the cortex primarily in the theta-band, and only a weak influence from the nucleus accumbens to the cortex in the alpha-band was present. Furthermore, we showed that this connectivity was already present in anticipation of visual stimulation, and increased during stimulus processing. We therefore suggest that theta-band oscillations serve the active exchange of stimulus information. Second, cortical to nucleus accumbens connectivity is implemented by alpha-band oscillations. Alpha-band connectivity in the neocortex predicted activity in the nucleus accumbens, and was, just like theta activity, present in anticipation of a visual stimulus, but did not increase upon stimulus processing. In line with current ideas on the functional role of cortical alpha-oscillations this might be an inhibitory con, serving to inhibit ongoing processing in the nucleus accumbens. We conclude that theta- and alpha-band oscillations serve different functional roles in cortico-striatal communication.

Oscillatory coupling between the nucleus accumbens and the frontal cortex has been previously found in humans during reward anticipation and reward processing. Cohen and colleagues showed coherence between nucleus accumbens and frontal cortex in the theta-band upon reward processing [13] and during reward anticipation [16]. Earlier, Cohen et al. found preliminary evidence for bidirectional communication between nucleus accumbens and frontal cortex during reward anticipation and reward processing, but did not investigate frequency-specificity [45]. Here we extend these findings by showing that connectivity between the nucleus accumbens and the cortex is implemented by oscillatory activity. This connectivity is present without expectation of reward or feedback, and is present both in anticipation of visual stimulation and during stimulus processing. Our findings are not likely to be explained by pickup of activity originating in the nucleus accumbens. In order to be measurable in the EEG an active neural population needs to be in an open field configuration [46]. The nucleus accumbens mostly consists of medium spiny neurons (e.g. [14,47]), which is a closed field configuration and it thus does not produce electric currents that can be measured as potential differences at the scalp level.

The strong influence from the nucleus accumbens to the neocortex might be surprising, given the polysynaptic connections from the striatum to the neocortex via the globus pallidus, subthalamic nucleus and the thalamus [17–19,48,49] and the hippocampus [50,51]. In contrast, the striatum receives monosynaptic input from the prefrontal areas [17]. Therefore one might expect a stronger influence from the neocortex to the striatum than the other way around. We, however, found that the control from nucleus accumbens over the cortex was about equally strong as the control from the cortex over the nucleus accumbens. In rats, McCracken and Grace have found that high-frequency stimulation of nucleus accumbens resulted in stronger low-frequency activity (0.5–4 Hz) and beta-activity (13–30 Hz) in orbitofrontal cortex [52,53], which also speaks to a strong, causal influence from nucleus accumbens to neocortex. Moreover, it was recently shown in monkeys that the striatum (here specifically the caudate nucleus) exerts a stronger influence in the beta-band (12–30 Hz) on the frontal cortex than the frontal cortex does on the striatum [54]. Likewise in humans, Smolders and colleagues have shown that deep brain stimulation of electrodes dorsally adjacent to the nucleus accumbens resulted in higher phase stability in the theta range over frontal scalp electrodes in OCD patients compared to when stimulation was turned off [55]. Our findings match these observations and also suggest that the polysynaptic connections from nucleus accumbens to the neocortex allow for a strong, causal influence.

We found a significant frequency-by-directionality interaction. In subsequent posthoc tests we found that this interaction indicated, that connectivity between NAc and Cortex was stronger in the theta than the alpha band, and also that there was a stronger difference between theta and alpha band Granger causality for the NAc-to-cortex than for the cortex-to-NAc direction. This, however, does not mean that theta oscillations exclusively subserve bottom-up processsing (from NAc to cortex). We tested this question by a permutation test and tested whether theta-band Granger causality is stronger in the NAc-to-cortex than in the cortex-to-NAc direction. Theta connectivity did not differ between directions. Our results thus suggest that the NAc is primarily, but not uniquely, employing theta oscillations to communicate with the cortex, probably reflecting an exchange of stimulus information (cf. [13,14,16]) as well as stimulus evoked activity.

While the theta-band connectivity might reflect a bi-directional exchange of stimulus information, we propose that alpha-band connectivity represents inhibitory control originating in the frontal cortex to actively suppress striatal processing. Functionally, this would be well in line with the recent view on cortical alpha-band oscillations (cf. [56–61]). Further substantiating this idea, it has been shown that electrical stimulation of the frontal cortex exerts strong inhibition onto striatal processing via hippocampal and thalamic pathways [62]. In order to verify these hypotheses on the differential functional roles of theta- and alpha-band oscillations, future studies could test how alpha- and theta-band connectivity are modulated in response to stimuli with different behavioral relevance. In our design, behaviorally irrelevant stimuli were simultaneously presented with relevant stimuli. Presentation of behavioral irrelevant and relevant stimuli in isolation will allow investigating the functional roles of theta- and alpha-band connectivity in more detail. We hypothesize that upon detection of a behavioral relevant stimulus, the striatum allowing for gating of the stimulus to prefrontal cortex, reflected by increased cortitical to nucleus accumbens theta-band phase synchrony. In contrast, presentation of behavioral irrelevant stimuli should elicit an increase in alpha-band connectivity between the cortex and the nucleus accumbens in order to prevent information about the stimulus to be exchanged.

It has recently been found that the nucleus accumbens is actively involved in gating visual information between neocortical regions. This was demonstrated in an fMRI study showing that the nucleus accumbens actively modulates frontoparietal connectivity during a visual attention task comparable to the one employed here [23,42]. A similar striatal modulation of corticocortical connectivity has been found in response to prediction errors [63]. Similarly we also found that the nucleus accumbens modulates frontoparietal connectivity as assessed by alpha band coherence. This is well in line with computational models, which predict that the striatum is involved in stimulus selection [20–22]. Concluding, our findings show the nucleus accumbens, the frontal and the parietal cortex constitute a functional entity during visual attention tasks, which are employing different frequency-bands for directional communication. In accordance with recent literature, this network might be involved in stimulus selection and processing [21,23,42].

The deep brain electrodes were implanted for clinical reasons to treat OCD, depression and addiction symptoms. Although we analyzed the most ventrally implanted electrodes, extending into the nucleus accumbens (see Methods), clinical effect in OCD patients are achieved by stimulation more dorsally, in the ventral part of the anterior limb of the internal capsule (vALIC), and not directly in the nucleus accumbens [3]. Deep brain stimulation of the vALIC modulates white matter tracts connecting basal ganglia to prefrontal and orbitofrontal cortical areas, which are likely to influence the interaction of cortex and the nucleus accumbens.

We investigated a mixed patient population of OCD, depression and drug addiction, which raises the question how our findings translate to the healthy brain. For example, OCD patients have been found to exhibit hyper-connectivity between the nucleus accumbens and prefrontal cortex (e.g. [64]). However, our results on the mixed group of participants suggest that our effects are not restricted to OCD patients, but extend to patients suffering from other pathologies. Also, we found no pathologic-specific differences between the patient groups, thus the found mechanism might be universal and not pathological. In addition, the task that the patients performed was kept free from pathological symbols or images to avoid symptom provoking stimulations. Moreover behavioral performance did not differ between patients and healthy subjects, who participated in an earlier study with a modified, slightly harder version of the task [24]. We therefore think that it is likely that similar corticostriatal connectivity patterns are present in healthy individuals as well.

In conclusion, our results shed new light on how functional connectivity in cortico-striatal networks is implemented by neuronal oscillations. Theta- and alpha-band oscillations reflected connectivity in different directions, and served different functional purposes. In line with recent investigations [23,42], we propose that the striatum actively gates behavioral relevant information to the neocortex during visual attention, here mediated by theta-oscillations, while behaviorally irrelevant information becomes suppressed as reflected by strong alpha-band connectivity. In future studies, the behavioral relevance of the spectrotemporal dynamics of cortical to nucleus accumbens connectivity could investigate this further by presenting distracters as well as target stimuli, which allows to specifically study the dynamics associated with gating of behaviorally relevant information.
